# The biomedical knowledge graph of symptom phenotype in coronary artery plaque: machine learning-based analysis of real-world clinical data

**DOI:** 10.1186/s13040-024-00365-1

**Published:** 2024-05-21

**Authors:** Jia-Ming Huan, Xiao-Jie Wang, Yuan Li, Shi-Jun Zhang, Yuan-Long Hu, Yun-Lun Li

**Affiliations:** 1 First School of Clinical Medicine, Shandong University of Traditional Chinese Medicine, Jinan, 250355 China; 2 Department of Cardiovascular, Affiliated Hospital of Shandong University of Traditional Chinese Medicine, Jinan, 250014 China; 3 Precision Diagnosis and Treatment of Cardiovascular Diseases with Traditional Chinese Medicine Shandong Engineering Research Center, Jinan, 250355 China

**Keywords:** Coronary artery plate, Biomedical knowledge graph, Symptom phenotypes, Machine learning, Network analysis, Clinical decision support

## Abstract

A knowledge graph can effectively showcase the essential characteristics of data and is increasingly emerging as a significant means of integrating information in the field of artificial intelligence. Coronary artery plaque represents a significant etiology of cardiovascular events, posing a diagnostic challenge for clinicians who are confronted with a multitude of nonspecific symptoms. To visualize the hierarchical relationship network graph of the molecular mechanisms underlying plaque properties and symptom phenotypes, patient symptomatology was extracted from electronic health record data from real-world clinical settings. Phenotypic networks were constructed utilizing clinical data and protein‒protein interaction networks. Machine learning techniques, including convolutional neural networks, Dijkstra's algorithm, and gene ontology semantic similarity, were employed to quantify clinical and biological features within the network. The resulting features were then utilized to train a K-nearest neighbor model, yielding 23 symptoms, 41 association rules, and 61 hub genes across the three types of plaques studied, achieving an area under the curve of 92.5%. Weighted correlation network analysis and pathway enrichment were subsequently utilized to identify lipid status-related genes and inflammation-associated pathways that could help explain the differences in plaque properties. To confirm the validity of the network graph model, we conducted coexpression analysis of the hub genes to evaluate their potential diagnostic value. Additionally, we investigated immune cell infiltration, examined the correlations between hub genes and immune cells, and validated the reliability of the identified biological pathways. By integrating clinical data and molecular network information, this biomedical knowledge graph model effectively elucidated the potential molecular mechanisms that collude symptoms, diseases, and molecules.

**Supplementary Information:**

The online version contains supplementary material available at 10.1186/s13040-024-00365-1.

## Introduction

There is mounting evidence that adverse cardiovascular events in patients with chronic ischemic heart disease are linked to the overall burden of atherosclerosis [1–3]. Coronary artery stenosis caused by myocardial ischemia is a common manifestation of coronary artery disease (CAD). Despite secondary preventive treatment, ischemic heart disease remains the leading cause of mortality and morbidity, with a high incidence of cardiovascular events [4]. While plaques typically do not rupture in the early stages of percutaneous coronary intervention (PCI) treatment, the risk in subsequent years still largely stems from coronary artery disease [5]. Early diagnosis and treatment of coronary atherosclerosis can thus significantly alleviate the disease burden of patients.

The symptom phenotype, which includes symptoms and signs, reflects the clinical characteristics of diseases and plays a vital role in disease diagnosis and treatment. In clinical practice, doctors mainly rely on the symptom information provided by patients to diagnose CAD. Junior doctors with limited clinical experience often rely on causal knowledge of the disease to make diagnoses, while senior doctors rely more on clinical experience, including the recollection of specific clinical cases [6]. Thus, the investigation of symptom combinations in patients with specific diseases as symptom phenotypes is of utmost importance. However, most clinical guidelines tend to describe more common symptoms in disease populations rather than at the individual level [7].

With the progress of technology, high-throughput sequencing and other techniques have been widely used in clinical research, but the potential molecular mechanism of symptom phenotypes has not been widely investigated; in particular, the use of nonspecific symptoms, such as fatigue, dizziness, headache and other symptoms, is still a challenge in the diagnosis and treatment of patients with CAD. Nonspecific symptoms are also part of the disease, but they are not enough to explain the pathological theory of persistent discomfort. One of the reasons why it is difficult to carry out this test is that the symptoms of these patients are clinical, and it is difficult to combine clinical information with experimental data effectively. Most of the existing common methods are to establish a multiplex network of clinical information and experimental information. Random walk with restart (RWR) and other classic algorithms are used to measure the importance of nodes [8, 9].

In modern times, the storage and transmission of clinical information and molecular biological information have become increasingly convenient. This has facilitated the integration and advancement of medical knowledge at various levels. As a visual representation of information structure, knowledge graphs are increasingly being utilized in the medical field. A medical knowledge graph can encompass a vast array of disease symptom characteristics and molecular biological characteristics. It offers broader coverage of entities and a wider range of semantic relations. Consequently, it serves as a valuable foundation for training machine learning models [10, 11]. Moreover, with the wide application of machine learning algorithms, in-depth mining technology for network graph information is constantly being developed [12–14].

The concept of knowledge graphs was introduced by Google in 2012 with the initial purpose of optimizing search engine results and enhancing user search quality and experience. Essentially, a knowledge graph is a technical approach that utilizes graph models to describe knowledge and model the relationships among entities in the world [15]. It is a symbolic representation of entities and their relationships in the objective world, aiming to achieve structured semantic knowledge storage.

The knowledge generated from medical activities is vast, encompassing not only the explicit knowledge found in patients' clinical diagnosis and treatment records but also the implicit knowledge governed by the micro mechanisms of the human body. Patient data constitute an essential part of the information required for clinical decision-making, along with the understanding of etiology, pathological processes, and the effectiveness of drugs or other therapeutic measures [16]. With the exponential growth of biomedical knowledge [17], there is a need to integrate medical clinical knowledge into microbiological systems to facilitate multidimensional representations of medical knowledge. The process of knowledge discovery and translation into practice involves extracting and isolating knowledge units from information sources and establishing appropriate representation models to achieve knowledge correlation.

**Fig. 1:**
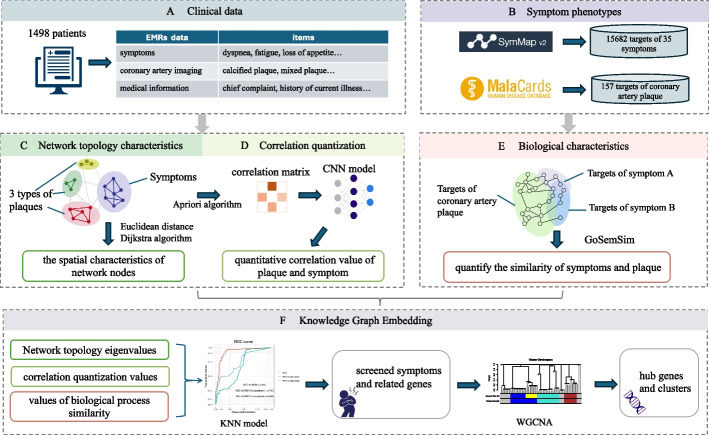
Association mining between plaque characteristics and symptom phenotypes: **A** Electronic medical record data from 1498 clinical patients were utilized to extract symptoms, coronary artery imaging, and medical information. **B** Symptom phenotypes and relevant targets of coronary artery plaque were collected from the SymMap and MaleCards databases. **C** A network was established with symptoms and three types of plaques as nodes, followed by network topology analysis. **D** A correlation matrix between symptoms and plaques was constructed, and a CNN model was trained to obtain correlation values. **E** Biological process similarity between symptoms and plaques was computed in PPIN. **F** The KNN model was used to screen symptoms and related genes, and hub genes were obtained using WGCNA

Therefore, as shown in Fig. 1, based on real-world electronic medical records, this study obtained information on symptoms and coronary artery plaque combined with relevant protein‒protein interaction information. Machine models and algorithms such as convolutional neural networks (CNNs) and K-nearest neighbors (KNNs) have been used to analyze clinical-molecular biomedical knowledge graphs and explain the clinical characteristics and internal mechanisms of different plaques from many perspectives.

## Materials and methods

### Data preparation

#### Clinical data

Clinical medical records were collected from 1498 patients who underwent coronary computed tomography angiography at the Affiliated Hospital of Shandong University of Traditional Chinese Medicine from August 2014 to June 2019. Demographic information, current symptoms, coronary artery imaging, and blood test results were collected from electronic medical records. Symptoms were standardized using Medical Subject Headings (MeSH). The characteristics of coronary artery plaques, including calcified plaque, noncalcified plaque, mixed plaque, and no plaque, were recorded. This study excluded plaques treated with coronary artery stents. Quality control was performed by a chief cardiovascular physician. In quality control, the physician is responsible for evaluating records, and the criteria for including patients are as follows:

The inclusion criteria were as follows: 1) Patients who had undergone chest spiral CT scans to obtain coronary artery images. 2) The images included the coronary arteries, left main trunk, left anterior descending branch, left circumflex branch, and right main trunk.

The exclusion criteria were as follows: 1) had a history of coronary artery bypass grafting. 2) Presence of myocardial bridging. 3) Poor image quality, including respiratory misalignment artifacts, discontinuities, and slice thicknesses greater than 1 mm.

#### Data on symptom phenotypes

The SymMap database contains various types of phenotypes related to diseases, including 1148 symptom terms and associated targets. It is currently being utilized in the research and development of new drugs, particularly natural drugs, for various chronic diseases, such as coronary artery atherosclerosis, Alzheimer's disease, and chronic atrophic gastritis [18–21]. The MalaCards human disease database integrates annotated disease information from 68 data sources, including symptoms, therapeutic drugs, and genes associated with diseases. It has been widely applied in genomic data annotation and drug repurposing model construction [22–26].

We sorted 35 symptoms from the electronic medical records data and collected the related targets from the SymMap (version 2.0) database [18] to establish the symptom phenotypes database. In addition, the related targets of coronary artery plaque were collected from the MalaCards database [22].

#### Protein‒protein interactions

The complexity of the physiological and pathological states of the human body originates from the functional and regulatory interactions between proteins, new protein interactions are constantly being discovered, and information is still dispersed in different database resources and experimental papers. The STRING [27] database systematically collects and integrates protein-protein interactions, including physical interactions and functional associations, all of which undergo strict scoring selection and can be used for the analysis of disease pathogenesis and the efficacy analysis of targeted drugs [28–31].

To obtain information about the interaction between symptom phenotypes and coronary plaque target proteins, we used *Homo sapiens* protein network data in the STRING (version 11.5) database to establish a protein‒protein interaction network (PPIN). The PPIN contains 12371 nodes and 2283976 edges with a combined score > = 400.

#### Set up network graphs

By collating symptom information and coronary artery plaque information from 1498 patients' electronic medical records, a clinical feature network was established. A network was constructed with 35 symptoms and 3 plaque properties as nodes. If two nodes appear in the same patient, they are connected as edges, with the number of patients in which this connection appears serving as the weight of the edge. This network comprises 608 edges.

### Knowledge fusion

#### Network topology characteristics

To comprehensively analyze the correlation information between the molecular network and clinical data, after merging the clinical feature network with the PPIN, we used the Dijkstra algorithm [32] to calculate the shortest distance between nodes. Combined with the Euclidean distance, we quantify the spatial characteristics of network nodes from point-to-point and point-network perspectives.

#### Correlation quantization

First, in the clinical-PPIN composite network, the Apriori algorithm [33] is used to calculate the lift value of coronary artery plaque and symptom phenotypes, which is used to quantify the correlation value between each item as the value of the plaque-symptom matrix. Each patient has a corresponding matrix, which contains all symptoms and plaque types in the dataset, while the data in this matrix include only the symptoms present in that patient. Because symptoms tend to aggregate specifically with different plaques, the local receptive fields of CNN models can sensitively capture local correlations in the matrix. The translation invariance of CNNs can also identify common features among matrices of different patients. The hierarchical structure filters out edge symptom phenotypes at lower levels and completes the identification of plaque feature symptom phenotypes at higher levels. Therefore, we used the actual symptom phenotypes and plaque properties of the patients in the medical records as the standard and used the CNN model to train the correlation matrix to establish the model. Then, the quantitative correlation value is determined by using the training model.

#### Biological characteristics

There are complex biological interactions in the molecular network. Nodes in PPINs often have different weights in biological processes. Gene Ontology (GO) biological process semantic similarity (GoSemSim) [34, 35] can return an association value between two genes after inputting them. Therefore, we used GoSemSim to quantify the similarity of different protein sets involved in biological processes and calculated the GoSemSim values of symptoms and plaques.

#### Knowledge graph embedding

The above analysis revealed clinical and molecular features across multiple dimensions. The basic assumption of the KNN model is that similar samples are close to each other in the feature space, effectively adapting to data distributions without requiring complex data transformations, thus capturing correlations between features well. Through appropriate feature selection and parameter tuning, KNN plays a role in handling multidimensional data, achieving classification filtering of data points. Additionally, predictions based on local neighborhoods can effectively capture the local structure of plaque and symptom data. Therefore, we use the KNN-A model for model training. The value of the RWR algorithm is taken as the judgment. We set the network topology feature value, correlation quantization value and biometric value as input information and trained with 10 cross-verifications [36] with K = 6.

To verify the necessity of model input information, two comparative models were constructed in this study. KNN-B takes the network topology eigenvalues and correlation quantization values as input information, and KNN-C takes biometric values of biological process similarity as input information. Both models used the KNN with the same set.

In addition, to further verify the wide applicability of the KNN model under this setting, first, we utilize gradient boosting decision tree (GBDT) and Bayesian network (BN) models to handle the association data between symptoms and plaques and train them with 10-fold cross-validation to compare their effectiveness with that of the KNN model. Additionally, we collected the electronic medical records of 2055 patients with hypertensive nephropathy using the same data processing method with the KNN model to analyze the relationships between hypertensive nephropathy and different symptoms and medications.

### Correlations between symptom phenotypes and clinical features

Using the KNN model, we screened the symptom phenotypes and related genes associated with three types of plaques. To further analyze the inherent associations between chronic diseases and blood parameters, we utilized weighted correlation network analysis (WGCNA) [37] to identify highly collaborative hub genes associated with phenotypes. WGCNA first calculates the weighted correlation coefficients between any two genes, that is, the N power of the gene correlation coefficient, ensuring that the connections between genes in the network follow scale-free properties. Then, gene selection is achieved through a threshold to obtain hub genes. Next, a hierarchical clustering tree was constructed based on the correlation coefficients between genes to identify modules where genes with similar patterns were grouped into different branches. Furthermore, the degree of association between genes within modules and phenotypes was measured using Pearson correlation coefficients. WGCNA was implemented using the *WGCNA* R package (version 1.72-5).

### Pathway enrichment analysis

To determine the biological processes involved in each gene set, Kyoto Encyclopedia of Genes and Genomes (KEGG) enrichment analysis and GO term enrichment analysis were used. A hypergeometric distribution was used, and a *P* value < 0.05 was considered to indicate a significant difference.

### Validation of the hub genes

The GSE28829 [38], GSE97210 [39], GSE104140 and GSE109048 [40] datasets were used to validate the expression of the hub genes. These four datasets contain RNA-seq data from plaque patients. The samples for these four studies were all derived from human subjects, including patients with early intimal thickening of atherosclerotic plaques and those with late-stage fibrous cap formation, as well as from healthy individuals, totaling 124 samples. The datasets underwent rigorous quality control and processing. Within each dataset, the expression data of the samples were balanced. Differential expression analysis was performed using the GEO2R tool to obtain differentially expressed genes in the four sample groups compared to their respective control groups (P < 0.05). All analyses were performed using the default settings of GEO2R. Gene expression was measured using the logFC value, and the effectiveness of the hub genes divided by WGCNA was evaluated using receiver operating characteristic (ROC) curves.

### Evaluation of immune cell infiltration

Pathway enrichment analysis revealed that the generation and growth of plaques are closely related to inflammation. To further confirm these findings and explore the immune cells involved in this pathological process, we used the GSE120521 [41] dataset and conducted analyses using the *xCell* (version 1.1.0) R package [42] and Cibersortx algorithms [43]. GSE120521 data were derived from RNA-seq of plaque tissue from patients. The permutation setting for the CIBERSORTx algorithm was set to 100. xCell analysis was performed using the preset function xCellAnalysis to calculate the enrichment scores of genes for each cell type, which were then converted into linear proportions while reducing dependencies between highly correlated cell types. These analyses allowed us to investigate the characteristics of the gene expression profiles of different immune cells. Additionally, we evaluated the correlation of genes related to immune cells in the internal environment using the immune score, stroma score, and microenvironment score.

## Results

### Symptom phenotypes and plaque association network graph

**Fig. 2:**
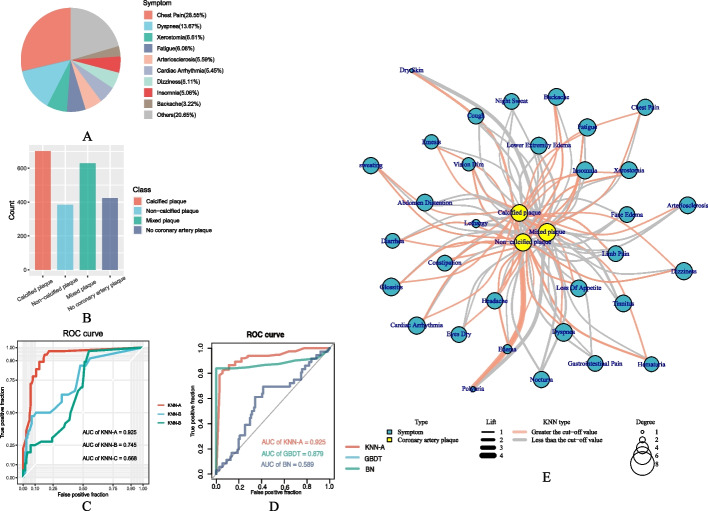
The association network between symptoms and plaques: (**A**) the symptom distribution of patients, (**B**) the number of different kinds of plaques in patients, (**C**) the ROC curve trained by the KNN model, (**D**) the ROC curve trained by the KNN, GBDT and BN models, and (**E**) the network diagram of the relationship between symptoms and plaques. The color of the points indicates the symptoms or plaques, the thickness of the edges represents the lift value in the Aprori algorithm, the color of the edges represents the results of the KNN model, and the size of the points indicates the degree of nodes

To obtain clinically relevant symptom information, we manually converted the symptoms recorded in patients' electronic medical records into standard MeSH descriptions, resulting in a total of 35 symptom types. Among these, chest pain, dyspnea, and fatigue are common cardiovascular symptoms (Fig. 2A). We collected data from 424 patients with no coronary artery plaque and 1074 patients with coronary artery plaque, including patients with calcified plaque, noncalcified plaque, and mixed plaque (Fig. 2B). Using these data, we established a network linking symptoms and coronary artery plaques. We also collected information on symptom phenotypes and coronary plaque-related genes from the SymMap and Malacards databases and used these data to establish a protein‒protein interaction network (PPIN) using the STRING database.

### Model analysis of network

To analyze the information from the PPIN and the clinical characteristics of patients, we utilized the KNN model to combine and correct the biological and clinical features obtained from algorithms such as CNN, Dijkstra, and GoSemSim. The resulting AUC value of KNN-A was 92.5%, which was greater than the AUC values obtained from KNN-B and KNN-C (74.5% and 66.8%, respectively), as shown in Fig. 2C, indicating that the model was effective. Additionally, as shown in Fig. 2D, the efficacy of the KNN-A model surpassed that of GBDT (87.9%) and BN (58.9%). We further validated the model by analyzing data from patients with hypertensive nephropathy, resulting in an AUC of 91.3%, which was consistent with related studies [44–47]. This demonstrates the wide applicability of the training data and the KNN model used in this study.

The KNN model was used to comprehensively evaluate the correlation between symptoms and plaques, resulting in the identification of 3 plaque properties, 23 kinds of symptoms, 41 association rules, and 61 hub genes. Fig. 2E shows that common symptoms such as chest pain, dizziness, and backache are associated with all 3 plaque properties. Calcified plaque was found to be associated with most symptoms (20 in total), including common symptoms of long-term chronic diseases such as dry eyes, limb pain, and arteriosclerosis. Symptoms related to fluid metabolism, such as edema and hematuria, were found to have higher lift values with noncalcified plaque, indicating that these symptoms are not commonly associated with other plaque types.

### Mining the diversity of clinical features

In addition to analyzing the relationship between symptoms and plaques, we collected information on patients' comorbidities and blood laboratory test results to gain a comprehensive understanding of the clinical characteristics of coronary artery plaques. Using the 61 hub genes identified by the KNN model, we evaluated their correlation with the actual symptoms and plaque properties of patients and conducted WGCNA to explore the relationships between these genes and the clinical conditions of the patients.

**Fig. 3:**
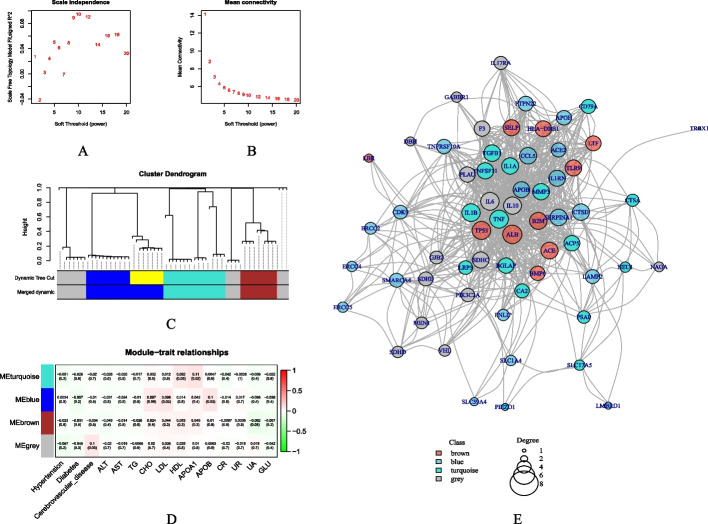
WGCNA results: (**A**) and (**B**) determine the soft threshold of the network recognition module; (**C**) the module division of the gene; (**D**) the relationship between the gene module and the clinical characteristics; (**E**) the network map of the gene module division. The color of the dot indicates the gene grouping, and the size of the point indicates the degree of the node

Based on the coefficients of the KNN model for each item, an association matrix between patients and proteins was established. After clustering and screening patients, the soft threshold of the network recognition module was determined to be 9 through network topology analysis (Fig. 3A, B). The genes were divided into four modules using hierarchical clustering and coexpression similarity (Fig. 3C, D). By calculating the correlation coefficient between gene modules and clinical characteristics, the genes screened by KNN were associated with the related indexes of lipid metabolism in patients. The genes in the blue group were concentrated in the serum lipid content, the genes in the turquoise group were concentrated in the serum apolipoprotein content, and the genes in the gray group were also associated with complicated cerebrovascular diseases (Fig. 3E).

### Molecular network mechanism of clinical symptoms

**Fig. 4:**
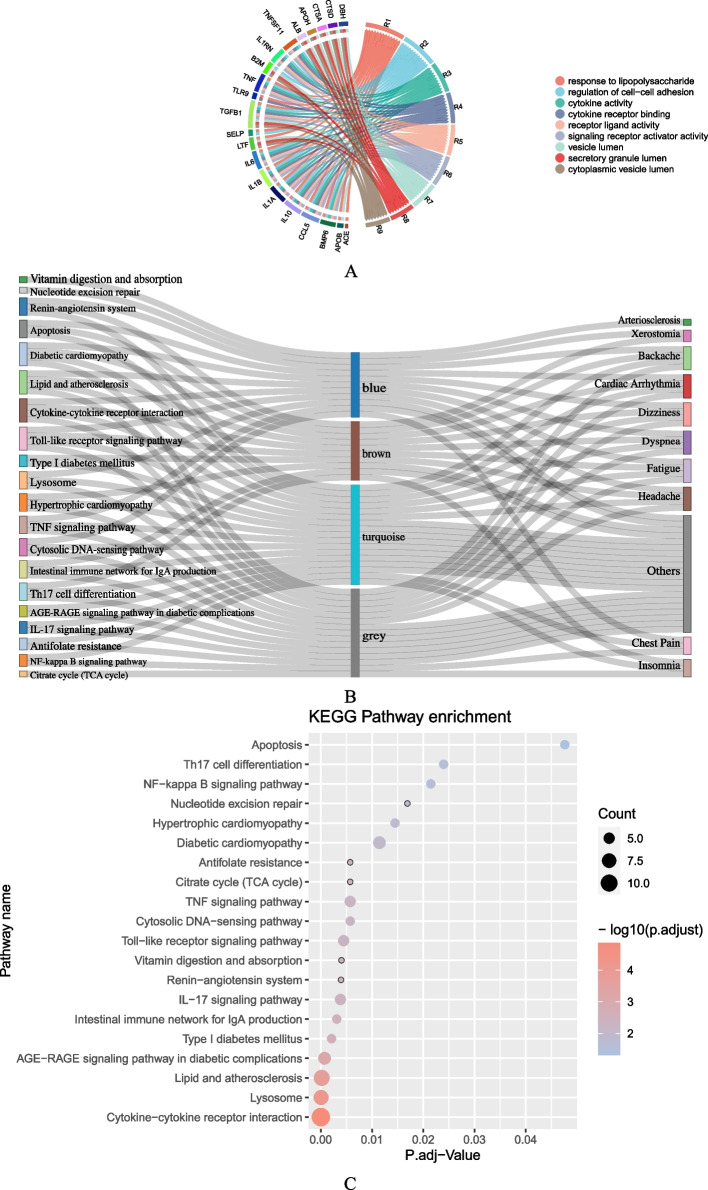
Pathway enrichment results: (**A**) GO enrichment results; (**B**) KEGG pathway, gene grouping and associated distribution of symptoms, and the colors in the middle column correspond to the grouping in WGCNA; (**C**) KEGG enrichment results

Functional enrichment analysis was performed for each group of genes categorized by WGCNA, and a functional annotation table was obtained. According to the Gene Ontology (GO) biological process category, each gene group was primarily involved in the response to lipopolysaccharide, regulation of cell–cell adhesion, cytokine activity, and cytokine receptor binding (Fig. 4A). These genes are closely related to the inflammatory response and energy metabolism. According to the KEGG analysis, 20 signaling pathways, including cytokine‒cytokine receptor interaction, lipid and atherosclerosis, the Toll-like receptor signaling pathway, and the TNF signaling pathway, which are associated with multiple gene groups and have the most extensive symptoms, were significantly enriched (Fig. 4B). The most significant KEGG pathway was cytokine‒cytokine receptor interaction (Fig. 4C).

### Expression characteristics of the hub genes

**Fig. 5:**
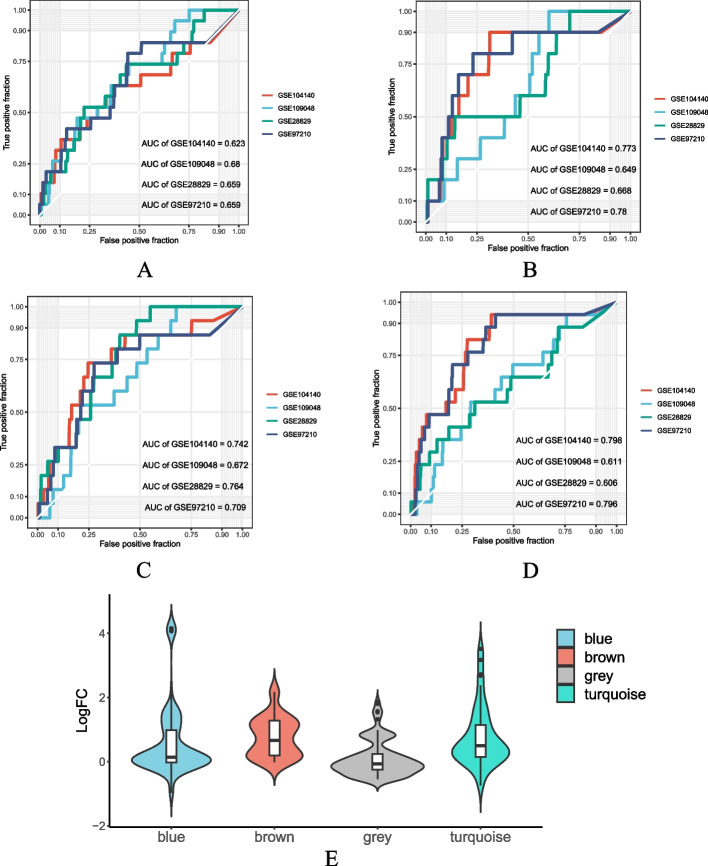
The expression characteristics of Hub genes: (**A-D**): The figures show ROC curves for the expression of four hub gene subgroups (blue, brown, gray, and turquoise) in different RNA-seq datasets (GSE28829, GSE97210, GSE104140, and GSE109048). The area under the curve (AUC) was used to evaluate the specificity of the hub genes. **E**: The distribution of logFC values of the hub genes in the RNA-seq dataset

According to the WGCNA of the clinical phenotype (Fig. 3E), the hub genes were divided into 4 subgroups: blue, brown, gray, and turquoise. As shown in Fig. 5 A-D, all four subgroups obtained higher AUC values in the RNA-seq dataset and showed abnormal expression compared to the control group (Fig. 5E).

### Immune cell infiltration

**Fig. 6:**
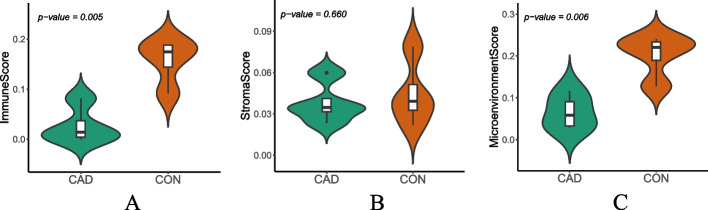
Immune cell infiltration: The results of the *xCell* package analysis, which is used to measure the correlation between genes and the plaque microenvironment. **A** Immune score, which measures overall immune activity in the plaque microenvironment. **B** Stroma score, which measures the degree of stromal cell infiltration in the plaque microenvironment. **C** Microenvironment score, which measures the overall level of interaction between immune and stromal cells in the plaque microenvironment

According to the results of the CIBERSORTx algorithm (uploaded to the supporting files), CD8 T cells, follicular helper T cells, M0 macrophages, M1 macrophages, and M2 macrophages were strongly correlated with plaque formation. There were significant differences in the immune score and microenvironment score compared with those of the control group, but there was no difference in the stroma score (Fig. 6A-C), suggesting that the percentage of immune cells in the plaque environment was greater and positively correlated with the expression of the hub genes.

## Discussion

In the early stage of plaque formation or stable plaque, the patient may have latent coronary heart disease, and myocardial ischemia may not occur until the cardiac load increases. As the plaque changes, typical symptoms of angina pectoris begin to appear, but the signs of angina pectoris are complex and have low specificity. Patients may experience anxiety, pale skin, chills or sweating, slight increases or decreases in blood pressure, an increased heart rate, and a systolic murmur in the apical area. The purpose of analyzing symptom characteristics is to improve doctors' ability to diagnose, predict, and treat diseases, deepen the understanding of disease mechanisms, and ultimately reduce the risk of adverse events. However, most symptoms are nonspecific.

Phenotyping can be performed based on clinical and molecular characteristics. Clinical phenotyping focuses on demographic information and laboratory tests, while molecular phenotyping emphasizes DNA, mRNA, proteins, and metabolites based on molecular characteristics. To comprehensively analyze this information and elucidate the relationship between plaque features and symptom phenotypes from multiple perspectives, we used the KNN model for knowledge graph embedding.

In this study, we utilized network analysis as the data source to establish a prescription feature network based on electronic medical records and a biological network based on protein‒protein interactions. We quantified the network information from multiple levels, including analyzing the biological similarity of different symptoms using GoSemSim, discussing the feature weights of symptom nodes and plaque nodes in the composite network using the Dijkstra algorithm and Apriori algorithm, and initially correcting the biological information and clinical information using the CNN model. Finally, we used the KNN model to train the knowledge graph model with multilevel information as input data to quantify the final association degree and determine the clinical symptoms and specific genes corresponding to the three plaques.

To study the mechanism of the symptom phenotype, we used WGCNA to construct a gene network, which formed a topological matrix and was divided into modules. Combined with pathway enrichment analysis, we found that among the three different plaque types, calcified plaques corresponded to the most extensive symptoms and were widely related to inflammation and lipid metabolism pathways. Moreover, lipid abnormalities in patients and other laboratory test results were associated with hub genes, while low-density lipoprotein levels were positively correlated with coronary artery calcification [48–50]. Immune cell infiltration analysis and existing studies have shown that coronary artery calcification mainly occurs in the intimal layer of blood vessels, and this process involves a variety of cells, such as macrophages, intimal cells, media smooth muscle cells, and fibroblasts [51]. Induced by a variety of calcium-stimulating factors, including interleukin enhancer-binding factor 3 (ILF3), smooth muscle cells inhibit alpha smooth muscle actin (α-SMA) and increase osteomodulin while migrating to plaques, which leads to phenotypic transformation of smooth muscle cells through their association with SMAD3 and TGFB1 signals and their interaction with BMP2 in vascular tissues [52–55]. On the other hand, macrophages exert their proinflammatory effects to further induce inflammatory cytokines such as IL-12 and IL-6, oxidize low-density lipoprotein in the arterial wall, and accelerate the decomposition of oxidized lipids by macrophages, resulting in increased plaque fragility [56, 57].

In the clinical context, inflammation has long been considered a primary driving factor in the formation of atherosclerosis and a key element in the development of vulnerable plaques [58]. The landmark Canakinumab Anti-Inflammatory Thrombosis Outcomes Study (CANTOS) [59] demonstrated, for the first time, a reduction in cardiovascular event recurrence with the use of Canakinumab, a monoclonal antibody targeting IL-1β. Studies on colchicine treatment for acute myocardial infarction have also underscored the role of inflammation in the pathogenesis of the disease [60]. Thus, accurate detection of vascular inflammation could help better stratify cardiovascular risk. Understanding the associations between plaque characteristics and symptom phenotypes can help clinicians understand the relationships between different symptoms and coronary artery plaques, thereby guiding clinical diagnosis and treatment decisions. This study makes full use of clinical data and molecular networks, integrating them through model construction, with the potential to provide more comprehensive information for disease diagnosis and treatment. This integrative research approach facilitates the connection between symptom manifestations, diseases, and molecules, offering new insights and methods for medical research.

The framework of this biomedical knowledge graph aims to establish a multilevel relationship between symptom phenotype and disease. By considering multiple symptoms rather than a single symptom, the patient population can be classified more specifically based on their symptom groups. Moreover, this study explored the molecular mechanism of the symptom phenotype, and the results were preliminarily verified at the RNA, biological pathway, and cellular levels, which provides a method for further investigation of the potential network mechanism of symptoms.

At the same time, there are also many shortcomings in this study. First, for the arrangement of clinical symptoms, the standard terms of symptoms in our biomedical database are quite different from the writing habits of Chinese medical records. We often encounter that there are no corresponding standard terms for the common descriptions in Chinese medical records, which are all excluded from this study. Second, in different types of plaques, calcified plaques tend to have a longer course of disease, which increases the likelihood that patients will experience abnormal blood pressure, blood sugar, or other target organ damage, which is the focus of this study. It is also a risk factor for potential bias. Third, the relevant evidence of hub genes comes from the algorithm simulation of clinical symptom data, and only a preliminary verification is carried out. In the future, we hope to further verify the hub genes at the cellular, animal, and clinical levels. The algorithm model will be further improved, and this model will be used to screen effective drug targets and better achieve fine individualized treatment services.

## Conclusion

This study presented a biomedical knowledge graph to comprehensively analyze disease characteristics and symptom phenotypes. This graph was used to investigate the characteristics and molecular mechanisms of coronary artery plaque features and symptoms, ultimately identifying the corresponding symptoms for three types of plaques. The findings of this study revealed that patients with calcified plaques exhibited more combined symptoms and complex biological processes. The underlying mechanism of the symptomatic phenotype was linked to the inflammatory response and biological processes related to lipid metabolism.

### Supplementary Information

Supplementary Material 1.

## Data Availability

The original electronic medical records data used in this study were uploaded to the supplementary materials. CNN model training was implemented in Python 3.7.2, and other data analysis was implemented in R 4.2.2.
